# A 1, 4-benzoquinone derivative isolated from *Ardisia crispa* (Thunb.) A. DC. root suppresses angiogenesis via its angiogenic signaling cascades

**DOI:** 10.1016/j.jsps.2023.101891

**Published:** 2023-12-01

**Authors:** Wen Jun Lim, Pit Foong Chan, Roslida Abd Hamid

**Affiliations:** Department of Biomedical Science, Faculty of Medicine and Health Sciences, Universiti Putra Malaysia, Serdang 43400, Selangor, Malaysia

**Keywords:** Benzoquinonoid, Anti-angiogenic, HUVECs, Cell invasion, Zymogram, Tube formation

## Abstract

The root hexane extract of *Ardisia crispa* (ACRH), which belongs to the Primulaceae family, has been reported to possess anti-inflammatory, chemopreventive, anti-arthritic, and antiangiogenic activities. In this study, we isolated a *p*-benzoquinone derivative, 2-methoxy-6-undecyl-1,4-benzoquinone (AC2), from ACRH and investigated its potential antiangiogenic activity in human umbilical vein endothelial cells (HUVECs) and zebrafish embryo models. Prior to this study, AC2 was characterized using ^1^H NMR spectroscopy and MS. AC2 significantly suppressed HUVEC proliferation in a time-independent manner, with an IC_50_ value of 1.35 ± 0.05, 1.15 ± 0.02, and 1.00 ± 0.01 µg/mL at 24, 48, and 72 h, respectively. AC2 also induced apoptosis in HUVECs and significantly suppressed their migration, invasion, and tube formation in a concentration-dependent manner. Additionally, AC2 significantly attenuated most of the analyzed protein markers, including pro-MMP-2, VEGF-C, VEGF-D, angiopoietin-2, endothelin-1, fibroblast growth factor (FGF)-1, FGF-2, follistatin, heparin-binding epidermal growth factor-like growth factor (HB-EGF), and hepatocyte growth factor (HGF) at all tested concentrations. Furthermore, AC2 significantly inhibited zebrafish embryo intersegmental vessels (ISVs), confirming its antiangiogenic role. In conclusion, AC2 exhibits a potential anti-angiogenic effect by suppressing several proangiogenic and growth factors. Further studies are needed to investigate their effects on other excessive angiogenic diseases.

## 1 Introduction

Angiogenesis, defined as the formation of new blood vessels from pre-existing ones, is a physiological process that occurs during embryonic development and later in the female reproductive tract of adult life, for a few days every month (Ribatti, 2013). This physiological process is in equilibrium and regulated by proangiogenic factors and angiogenesis inhibitors. Pathological angiogenesis, which occurs when there is an angiogenic switch, leads to either excessive or insufficient angiogenesis under certain conditions such as hypoxia, inflammation, and hyperalgesia, which in turn dramatically increases endothelial proliferation. Amongst the diseases related with excessive angiogenesis disorders are tumour angiogenesis (cancer), rheumatoid arthritis, diabetic retinopathy, atherosclerosis, multiple sclerosis, psoriasis, obesity, asthma and such (Warmke et al., 2018).

Despite the emergence of more anti-angiogenic agents available in the market with promising performance during the initial clinical phase, the long-term clinical benefits of these therapies for cancer treatment have been rather modest. The progression-free survival of these novel therapies has only improved for a few months, with no improvement in overall survival rates (Bellou et al., 2013). In addition, the high cost, serious adverse effects, and potential resistance development of existing antiangiogenic agents necessitate the identification of other novel, inexpensive, minimal side effects and effective anti-angiogenic molecules (Lu et al., 2016).

The development of new anti-angiogenic agents is based on the various molecular structures of natural phytochemicals (Kotoku et al., 2016). Furthermore, botanicals contain an elaborate range of organic chemical complexes that can act on multiple angiogenic pathways to reduce drug resistance through compensatory mechanisms (Sun et al., 2015). Thus, phytochemicals are currently being extensively examined to discover novel multiple target agents that can be scientifically transformed into therapeutic advantages (Gatne and Addepalli, 2013).

*Ardisia crispa* (Thunb.) A. DC. (Primulaceae) is broadly distributed in Asia, including the Himalayas, India, China, Japan, Indo-China, and Malay Archipelago (Perry and Metzger, 1980). Taxonomically wise, this plant, locally named as “mata itik” or hen’s eyes in Malaysia has been mistakenly identified for another species of similar morphology, *Ardisia crenata* (Raven & Wu, 1996). Both the leaves and roots of this plant have been used in traditional practices by the local villagers. The root is used as a decoction to treat fever, swelling, pain, and blood circulation (Perry and Metzger, 1980). Its leaves are crushed and applied at affected sites as an antidote for scorpions and snake bites (Muhammad & Mustafa, 1994). In Thailand, the root is mixed with other plants to treat women with dysmenorrhea (Chaweewan, 1995). In the Indo-China region, locals treat chest illnesses with plant root extract, while Taiwanese people use it as a diuretic and an antidote for poisons (Perry & Metzger, 1980).

**Fig. 1:**
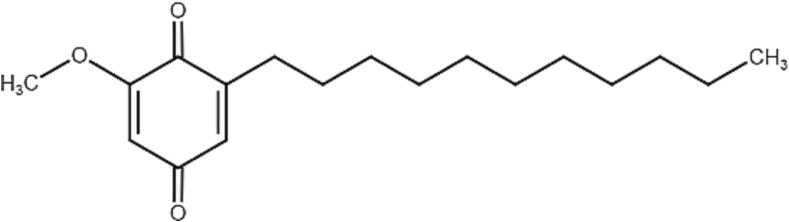
2-methoxy-6-undecyl-1,4-benzoquinone (AC2) (Roslida, 2004).

A benzoquinonoid derivative, 2-methoxy-6-undecyl-1, 4-benzoquinone (Fig. 1), has been previously isolated from ACRH. To date, there have not been many studies on this compound, except for its inhibition of acute inflammation and hyperalgesia (Roslida, 2004). We had previously reported multiple studies on the plant’s root hexane and its fractionated extracts enriched with this aforementioned compound, labelled as ACRH and QRF, respectively, including anti-inflammatory, anti-arthritic, antitumor promotion and antiangiogenic (Roslida and Kim, 2008, Roslida et al., 2009, Lau et al., 2009, Roslida et al., 2011, Sulaiman et al., 2012, Hamid et al., 2013, Hamsin et al., 2013, Hamsin et al., 2014, Yeong et al., 2013, Yeong et al., 2014, Yeong et al., 2015, Hamid et al., 2017, Wen Jun et al., 2019, Blin et al., 2021a, Blin et al., 2021b), However, little was known about the principal bioactive compound in ACRH and QRF that might also be responsible for its antiangiogenic property and the possible pathway involved. Therefore, in the current study, we isolated the compound and tested it in various *in vitro* angiogenesis and *in vivo* zebrafish assays, as well as in elucidating the potential protein targets responsible for the antiangiogenic effect of AC2.

## 2 Materials and methods

### 2.1 Chemicals and reagents

All chemicals used were of analytical grade and commercially available unless otherwise specified. Suramin hexasodium salt, sunitinib, recombinant human vascular endothelial growth factor (VEGF), and a zymogram kit were purchased from Abcam (UK) and Cosmo Bio (Japan), respectively. ApopNexin^TM^ FITC apoptosis detection kit; Milipore (USA), 3-(4,5-dimethylthiazol-2-yl)-2,5-diphenyl tetrazolium bromide (MTT) from Calbiochem (USA), BD BioCoat™ Matrigel™ Invasion Chamber and Matrigel Matrix were from BD Sciences (USA), Milliplex® Map Human Angiogenesis/Growth Factor magnetic Bead Panel 1 96-well plate kit from EMD Milliopore (USA).

### 2.2 Plant materials

**Fig. 2:**
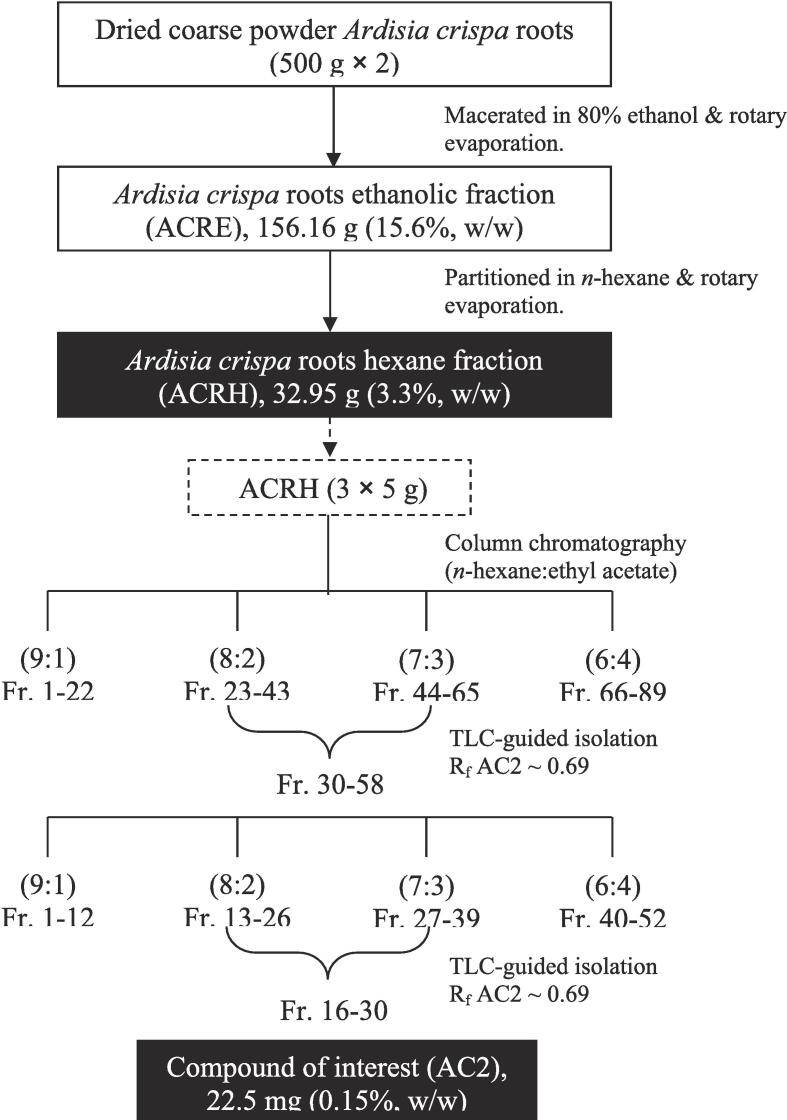
Schematic illustration of extraction and isolation of a bioactive compound, AC2 from *Ardisia crispa* root via column chromatography and TLC-guided isolation. Fr, Fraction.

The plant was obtained from Kelantan, Malaysia, authenticated, and deposited in the university herbarium (voucher specimen no. 20841). Isolation of the bioactive compound and its prior extraction from *Ardisia crispa* roots have been described previously (Hamsin et al., 2013) and are illustrated in Fig. 2.

### 2.3 Composition analysis by gas chromatography-mass spectrometry (GC–MS)

The chemical composition of the compound isolated from ACRH was analyzed using GC–MS, as described previously (Yeong et al., 2014). Briefly, GC–MS was performed using an Agilent model 5973 MSD gas chromatograph (Agilent Technologies, USA) fitted with a fused silica column, HP-5MS (30 m × 250 μm with a 0.25 μm film thickness, coated with phenyl-methylpolysiloxane. The mass spectrometer was operated in the electron impact ionization mode with an ionization voltage of 70 eV and a mass scan range of 50–550 aMu. The sample was first dissolved in hexane at five-fold dilution and centrifuged to remove particulates prior to injection into the GC–MS system. Two microliters of the sample were injected in splitless mode and carried by purified helium at a flow rate of 1 mL/min. When the injector port temperature was set to 250 °C, the oven temperature was gradually increased from 70 °C to 300 °C at a rate of 10 °C/min and held for 6 min, with a total run time of 29 min. Mass fragmentation data were obtained from the National Institute of Standards and Technology (NIST) library for compound identification. It was then compared with the data for the reference compounds from previous studies (Roslida, 2004, Yeong et al., 2014).

### 2.4 Characterization of isolated compound by proton nuclear magnetic resonance (^1^H NMR) spectroscopy

The isolated sample was dissolved in deuterochloroform (CDCl_3_) and transferred to a 5 mm NMR tube for analysis. The NMR spectra were measured and recorded on a Fourier Transform Nuclear Magnetic Resonance (FT-NMR) model Bruker-Advance III™ 400 MHz (Bruker, Germany), which was interfaced with TOPSPIN 2.1 software and pulse program zg30 for ^1^H NMR. The coupling constants (J) were quoted in Hertz (Hz), while the chemical shifts (δ) were reported in parts per million (ppm) and were relative to tetramethylsilane (0 ppm), with the residual solvent signal of deuterochloroform (CDCl_3_) of 7.26 ppm.

### 2.5 Cell culture

HUVECs (ScienCell, USA) were cultured in a complete endothelial cell growth medium (EGM) consisting of 1 % endothelial cell growth supplement (ECGS), 5 % fetal bovine serum (FBS), and 1 % penicillin/streptomycin. Cells were propagated as monolayers at 37 °C in a 5 % CO_2_ atmosphere. Only cell passages–3–6 were used in this study to avoid senescence stage of the cells (Bouis et al., 2001).

### 2.6 Cell proliferation assay

A colorimetric assay using MTT reagent was performed to determine the viability and proliferation of human umbilical vein endothelial cells (HUVECs). The assay was performed according to the method described by Mosmann, (1983) Briefly, HUVECs were seeded in triplicate at a density of 4 × 10^3^ cells/well in 96-well plates and incubated at 37 °C and 5 % CO_2_ overnight for recovery. The following day, the cells were treated with various concentrations of AC2 (ranging from 0.15 to 20 µg/mL) and incubated for 24–72 h in the dark at 37 °C and 5 % CO_2_. Next, 20 µL MTT solution was added to each well and incubated for an additional 2 h under the same conditions. The content in each well was aspirated, and 100 µL of DMSO was added to each well to solubilize the purple formazan crystals. The plate was analyzed using a microplate reader (Biochrom, UK) at a wavelength of 570/630 nm. The number of surviving cells after treatment with AC2 at each time interval was expressed as a percentage. The IC_50_ values of AC2 against HUVECs were obtained from log concentration–response curves.

### 2.7 Cell apoptosis assay

To confirm that the reduction in viable cells was due to the antiproliferative effect of the treatments instead of their cytotoxicity, the cell apoptosis profile was assessed using the ApopNexin^TM^ FITC apoptosis detection kit (Millipore, USA), following the protocol recommended by Hingorani et al. (2011). HUVECs (1 × 10^4^ cells/well) were seeded in a 24-well plate and incubated for 24 h at 37 °C and 5 % CO_2_. Cells were treated with various concentrations of AC2 (0.1, 1.0 and 10.0 µg/mL) and incubated for 24 h at 37 °C and 5 % CO_2._

After harvesting the cells at the end of the experiment, they were transferred into a chilled culture tube and centrifuged at 400 × *g* for 5 min, and the supernatant was discarded. After washing and centrifugation twice with ice-cold PBS, cells were suspended in ice-cold Binding Buffer at a concentration of 10^6^ cells/mL. Annexin V-FITC (3 µL) was mixed well with 200 µL cell suspension prior to the addition of propidium iodide (PI) reagent (2 µL) into the Annexin V-FITC labeled cells. The cell suspensions were mixed and incubated for 15 min at room temperature in the dark. The samples were placed on ice prior to analysis.

The stained cells were examined using a flow cytometer (BD Sciences, USA) with a band-pass filter for Annexin V-FITC and PI at 530 nm and 600 nm, respectively. According to the manufacturer’s protocol, events falling in the Annexin V+/PI- region of the lower right quadrant were counted as early apoptotic cells, events falling in the Annexin V+/PI + region of the upper right quadrant were counted as late apoptotic cells, and events falling in the Annexin V-/PI- region of the lower left quadrant were counted as viable cells.

### 2.8 Cell cycle analysis

The cell cycle distribution of HUVEC was analyzed according to a previous study with slight modifications (Pozarowski and Darzynkiewicz, 2004). First, HUVECs (1 × 10^6^ cells/well) were seeded in a 6-well plate and incubated for 24 h at 37 °C and 5 % CO_2_. The medium was then discarded, and the cells were washed with ice-cold PBS prior to treatment with various concentrations (0.1, 1.0 and 10.0 µg/mL) of AC2 and incubated for another 24 h at 37 °C and 5 % CO_2._ Upon completion of the treatment, the cells were trypsinized and centrifuged together with the culture medium at 300 × *g* for 5 min and then washed twice with ice-cold PBS. The PBS was then discarded, and the cells were slowly mixed with ice-cold 70 % ethanol. The cells were then stored at −20 °C for overnight. Cell fixation with ethanol was used to increase the permeability of the cell membrane and to enhance the binding of propidium iodide (PI) to cellular DNA.

As PI is not a DNA-specific dye, all nucleic acids, including RNA, may be stained by PI. Thus, RNase treatment was necessary to ensure PI DNA staining. The ethanol was discarded, the fixed cells were washed with ice-cold PBS before centrifugation at 300 × *g* for 5 min, and the supernatant was removed. The cell pellet was suspended in 425 µL fresh PBS supplemented with 50 µL RNase A (1 mg/mL) and 25 µL PI (1 mg/mL). Staining was performed by incubating the cells at room temperature in the dark for 30 min. Cell cycle distribution was then examined by flow cytometry, and the number of events at each stage of the cell cycle was expressed as a percentage (%).

### 2.9 Cell migration/Wound healing assay

Endothelial cell migration is required for tumor angiogenesis; therefore, cell motility is of particular interest in the development of antiangiogenic therapy (Goodwin, 2007). The effect of AC2 on HUVECs migration was studied using a wound healing assay, as previously described (Guo et al., 2014). Briefly, cells were seeded in 6-well plates at 1 × 10^6^ cells/well in 2.0 mL medium and allowed to reach 90 % confluency. Subsequently, the cell monolayer was scratched with a sterile 200 μL micropipette tip, washed with PBS, and 2.0 mL fresh medium containing treatments were added. The wounds were photographed immediately and after 24 h of treatment with various concentrations (0.1, 1.0 and 10.0 µg/mL) of AC2 respectively. At 0 and 24 h, an inverted light microscope with a digital camera (Carl Zeiss, Germany) was used to record wound closure and was measured using Tscratch software (Hall and Brooks, 2001). The percentage of wound closure was calculated using the formula described by Aisha et al. (2013):

$$\%wound\;closure=\left(1-\frac{wound\;area\;at\;24\;h}{wound\;area\;at\;0\;h}\right)\times 100$$

An increase in the percentage of open wound area indicates the inhibition of cell migration.

### 2.10 Chemotaxis cell invasion assay

This method was described in accordance with previous studies by (Hulkower and Herber, 2011) with slight modification, using a commercialized invasion chamber (BD BioCoat ™ Matrigel ™ Invasion Chamber). The two compartments of the chamber were separated using an 8 µm pore size polyethylene terephthalate (PET) membrane with a thin layer of Matrigel basement membrane matrix. Following the manufacturer’s protocol, the insert was removed from −20 °C storage and allowed to reach room temperature. Culture medium (0.5 mL) was added to the interior of the inserts and bottom of the wells and incubated at 37 °C in a 5 % CO_2_ atmosphere for 2 h. After rehydration of the membrane, all media were carefully removed without disturbing the membrane layer. 0.75 mL of culture medium supplemented with 10 ng/mL VEGF, as a chemoattractant, was added to each well. The inserts were then carefully transferred to the wells using sterile forceps to ensure that no air bubbles were trapped underneath the membranes. 0.5 mL of cell-containing culture medium (2.5 × 10^4^ HUVEC) with different concentrations (0.1, 1.0 and 10.0 µg/mL) of AC2 was immediately added to each insert and incubated at 37 °C in a 5 % CO_2_ atmosphere for 22 h.

After 22 h of treatment, non-invading cells were removed from the upper surface of the membrane by gently scrubbing the inserts with a cotton-tipped swab. The inserts were rinsed with PBS before being fixed with absolute methanol for 10 min. The invading cells on the lower surface of the membrane were stained with hematoxylin and eosin. After rinsing twice with PBS, the inserts were allowed to air-dry. The insert was then placed on a glass slide and observed under a light microscope at 200 × magnification. Cell counting was facilitated by photographing the membrane in six fields using a cell counter.

Data were expressed as the percentage invasion of treated cells through the Matrigel matrix and membrane relative to the negative control, which contained only HUVEC with culture medium.

### 2.11 Tube formation assay

The formation of capillary-like tubules in vitro on the basement membrane matrix simulates many steps of the angiogenesis process *in vivo*, especially endothelial cell differentiation at the later stage of angiogenesis (Arnaoutova and Kleinman, 2010). The capacity of HUVECs to form tubular networks after AC2 treatment was evaluated in the current study using previously described methods, with slight modifications (Guo et al., 2014).

Prior to this experiment, a pre-chilled apparatus was used to avoid premature solidification of the Matrigel (BD Biosciences, USA) by thawing at 4 °C overnight. Matrigel (250 µL) was added to each well and incubated at 37 °C for 30 min to allow for solidification. HUVECs (4 × 10^4^/well/300 µL media) were seeded and treated with AC2 at various concentrations for 16 h (37 °C, 5 % CO_2_). Next, the cells were rinsed with 1 mL PBS, and the medium was removed. Tubular networks were quantified by examining their images at 200× magnification under an inverted microscope (Carl Zeiss, Germany). The images were analyzed using ImageJ software with the Angiogenesis Analyzer plugin for the quantification of tube networks (DeCicco-Skinner et al., 2014).

### 2.12 Zymogram assay

The expression of gelatinases in HUVEC was determined using a gelatin zymography kit (CosmoBio®, Japan), as previously described with slight modifications (Toth and Fridman, 2013). HUVEC were seeded in a 6 well-plate at a density of 1 × 10^6^ cells/well and incubated in complete medium for 24 h at 37 °C and 5 % CO_2_. The cells were then treated with 0.1, 1.0 and 10.0 µg/mL of AC2 in a medium without FBS, and the culture supernatant was collected after 24 h of treatment. The collected samples were mixed with an equal amount of sample preparation buffer (under non-reducing conditions) and incubated for 15 min at room temperature. The mixture was then loaded into a precast gelatin zymography gel and subjected to electrophoresis at a constant current of 15 mA. The gel was removed from the glass gel plate when electrophoresis was complete, incubated in washing buffer, and shaken at room temperature for 30 min. The gel was then incubated with reaction buffer at 37 °C for 24 h. After the enzymatic reaction, the gel was stained with a staining solution at room temperature for 30 min and destained with 5 % acetic acid in 30 % methanol until a clear band appeared against the blue background. The clear bands represented active gelatinase (MMP-2 and MMP-9) activities following a specific molecular weight and could be compared with the MMP markers provided. The band intensities were quantified by densitometric analysis using the ImageJ software.

### 2.13 Multipex immunoassay

The Magnetic Bead Panel 1 from Merck Millipore, designated as Milliplex® Map Human Angiogenesis / Growth Factor Magnetic Bead Panel 1, was used to detect and quantify 17 human angiogenesis and growth factor biomarkers, including Angiopoietin-2, Bone Morphogenetic Protein-9 (BMP-9), Epidermal Growth Factor (EGF), Endoglin, Endothelin-1, Fibroblast Growth Factor-1 (FGF-1), Fibroblast Growth Factor-2 (FGF-2), Follistatin, Granulocyte-Colony Stimulating Factor (G-CSF), Heparin Binding EGF (HB-EGF), Hepatocyte Growth Factor (HGF), Interleukin 8 (IL-8), Leptin, Placental Growth Factor (PLGF), Vascular Endothelial Growth Factor A (VEGF-A), Vascular Endothelial Growth Factor C (VEGF-C), and Vascular Endothelial Growth Factor D (VEGF-D, in HUVEC lysate, using the Luminex system and following the manufacturer's instructions.

To perform the assay, HUVECs were cultured in 100 mm cell culture dishes until they reached sub-confluence. Cells were treated with various concentrations (0.1, 1.0, and 10.0 µg/mL) of AC2 for 24 h at 37 °C and 5 % CO_2_. After treatment, the culture medium was discarded, and adherent cells were washed twice with cold PBS.

Next, approximately one mL of cold RIPA buffer (Thermo Scientific, USA) supplemented with a protease inhibitor cocktail (Nacalai Tesque, Japan) was added to the culture flask and kept on ice for 5 min with occasional swirling for uniform spreading. The addition of a protease inhibitor prevents proteolysis of the protein. The cell lysates were then collected using a cell scraper and transferred to a microcentrifuge tube. The cell lysates were centrifuged at 14,000 × g for 15 min to pellet the cell debris and were later analyzed using the Bio-plex systems (Bio-Rad, USA) according to the manufacturer's instructions.

### 2.14 Zebrafish angiogenic assay

In the current study, an angiogenic assay was conducted on *Tg(fli1:EGFP)^y1^* transgenic zebrafish using a slightly modified previously described procedure (Li et al., 2014). The zebrafish were maintained in a temperature-controlled room at 28 °C with a 14:10 h day/night cycle. The male-to-female ratio was set at 1:1, and zebrafish were randomly selected for mating and housed in breeding tanks. Embryos were collected from the breeding trap, washed thoroughly, and maintained in embryo water (E3) in an incubator at 28.5 °C in the dark. Dead or unfertilized embryos were removed periodically to prevent developmental delay in healthy embryos. The embryos were manually dechorionated with forceps 20 h post fertilization (hpf) prior to treatment with the test samples. The embryos were arrayed in 24-well plates at 10 embryos per well and incubated at 28 °C for 24 h in 1 mL of E3 + 0.003 % 1-phenyl 2-thiourea (PTU) containing the vehicle control (DMSO 0.5 %) and treatment solutions (AC2, suramin, and sunitinib). After drug treatment, the number of intersegmental vessels formed in each embryo was evaluated and imaged using fluorescence microscopy. The results are presented as the percentage of vessel growth, and the experiment was repeated thrice.

### 2.15 Statistical analysis

SPSS Statistics software (version 17.0) was used to analyze the data. Multiple pairwise comparisons between different groups via One-way ANOVA followed by post-hoc Tukey test were used with p < 0.05, considered significant.

## 3 Results

### 3.1 Extraction and isolation of AC2

Approximately 1 kg of the plant’s root yielded 32.95 g (3.3 %, w/w) dark brownish colored ACRH. Subsequently, column chromatography was used to further separate 15 g of ACRH, which eventually yielded 22.5 mg of AC2 (0.15 %, w/w) (Fig. 2). The isolated compound exhibited a similar R_f_ (0.71) as the reference compound (R_f_ = 0.69) and was a yellow, amorphous powder, which showed a visible yellow spot on the TLC plate and fluorescent pink spot when observed under 254 nm UV light. Upon heating, after spraying with 10 % H_2_SO_4_, the yellow spot turned bluish-black.

All observations in this study are consistent with those reported by Roslida (2004). A schematic diagram representing the flow of extraction and isolation of AC2 is shown in Fig. 2.

### 3.2 Chromatographic analysis and characterization of AC2

**Fig. 3:**
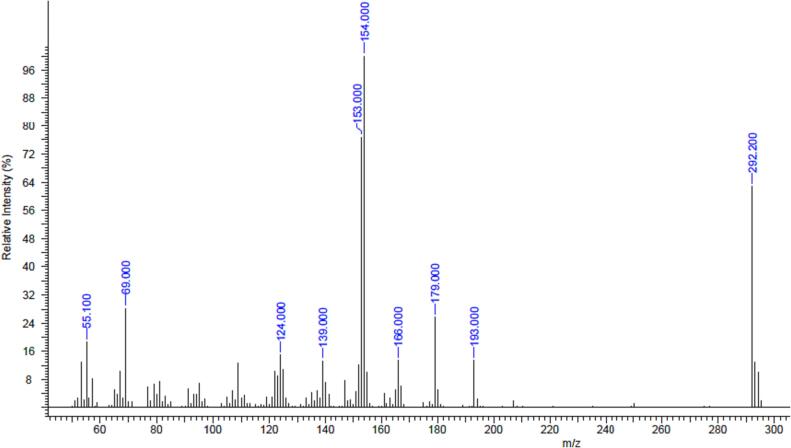
GC–MS spectrum of 2-methoxy-6-undecyl-1,4-benzoquinone (AC2).

The GC–MS spectrum of compound (AC2) was compared with the data reported by Yeong et al. (2014) for the same compound. The current MS data are in line with those obtained in a previous study (Yeong et al., 2014). The MS spectrum of AC2 (Fig. 3) shared similarities with the base peak at *m*/*z* 154 (1 0 0) and ion peak at *m*/*z* 292 [M^+^], which corresponded to the molecular formula of C_18_H_28_O_3_. The mass spectra of the isolated compounds in the current study and the reference, AC2, are summarized in Table 1.

**Table 1:** Mass spectra for the isolated compound in the current study and reference compound, AC2.

**Isolated compound**		**Reference compound (**Yeong et al., 2014**)**	
***m*/*z***	**Relative intensity (%)**	***m*/*z***	**Relative intensity (%)**
292	63 [M^+^]	292	58 [M^+^]
193	13	193	12
179	26	179	24
166	13	166	12
154	100	154	100
153	77	153	75
139	13	139	13
124	14	124	14
69	28	69	27
55	18	55	17

**Fig. 4:**
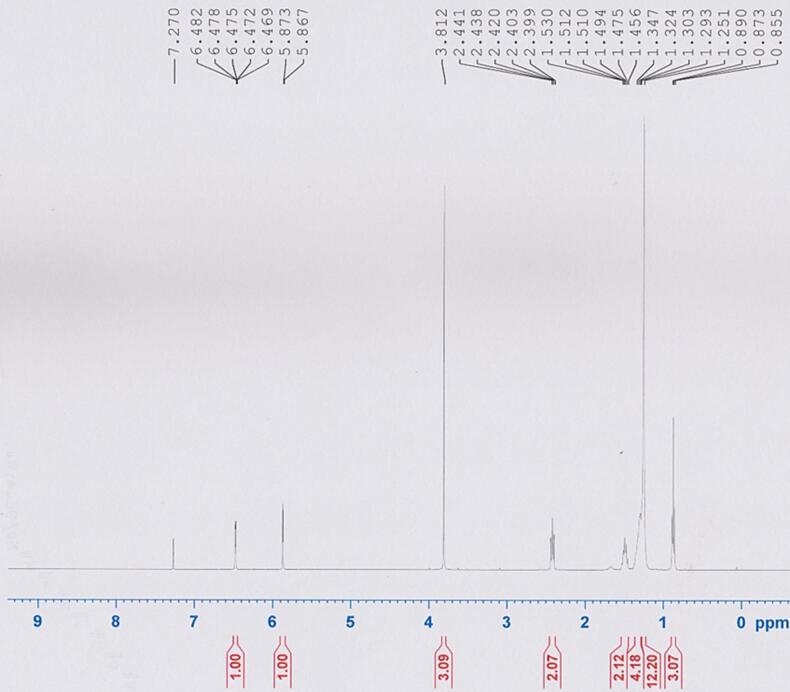
^1^H NMR spectrum of 2-methocy-6-undecyl-1,4-benzoquinone (AC2).

The characteristics of the isolated compound were further confirmed by ^1^H NMR analysis, which was in line with the data reported by Yeong et al. (2014). ^1^H NMR spectra (Fig. 4) are summarized in Table 2. The similarity between the isolated compound and AC2 was confirmed by ^1^H NMR spectroscopy, indicating that their chemical structures were matched.

**Table 2:** ^1^H NMR data of isolated compound.

H position	Multiplicity	Chemical shift (δ)
H-5	d (1H, *J* = 2.4 Hz)	6.48
H-3	d (1H, *J* = 2.4 Hz)	5.87
OCH3	s (3H)	3.81
H-1′	t (2H, *J* = 8.4 Hz)	2.42
H-2′	m (2H)	1.51
H-3′-H-10′	overlap (16H)	1.32–1.25
H-11′	t (3H, *J* = 6.8 Hz)	0.87

### 3.3 Antiproliferative, apoptotic and cell cycle distribution effects of AC2 on HUVECs

The anti-angiogenic activity of AC2 was initially evaluated based on its anti-proliferative and apoptosis-inducing effects in HUVECs. As presented in Table 3, AC2 exhibited potent suppressive effects on HUVEC proliferation within the range of 0.15–20 μg/mL for 24, 48, and 72 h. The IC_50_ values were determined to be 1.35 ± 0.05, 1.15 ± 0.02, and 1.00 ± 0.01 μg/mL, respectively. Concentration-dependent inhibition of HUVEC growth was observed, with the strongest inhibitory effect noticed at a concentration of 1.25 μg/mL, and a plateau was achieved at 2.5 μg/mL and above. It can be inferred that AC2 may restrict HUVEC proliferation within a limited range of concentrations. However, the antiproliferative activity of AC2 was not time-dependent, as prolonged treatment duration did not significantly affect the IC_50_ value.

**Table 3:** Percentage of HUVECs proliferation upon treatment with AC2 at 24, 48 and 72 h.

Concentration (µg/mL)	Growth control (% Mean ± S.E.M)		
	24 h	48 h	72 h
0	100.00 ± 0.00^a^	100.00 ± 0.00^a^	100.00 ± 0.00^a^
0.15	101.49 ± 2.45^a^	101.55 ± 0.67^a^	104.95 ± 0.79^a^
0.30	105.70 ± 0.79^a^	101.46 ± 1.26^a^	97.87 ± 1.58^a^
0.62	102.03 ± 0.93^a^	101.87 ± 2.04^a^	97.56 ± 0.43^a^
1.25	39.26 ± 4.38^b^	28.57 ± 1.51^b^	15.05 ± 2.28^b^
2.5	8.23 ± 0.16^c^	3.66 ± 0.01^c^	3.55 ± 0.05^c^
5.0	6.99 ± 0.12^c^	3.64 ± 0.04^c^	3.55 ± 0.01^c^
10.0	6.75 ± 0.11^c^	3.68 ± 0.03^c^	3.54 ± 0.06^c^
20.0	6.51 ± 0.07^c^	3.65 ± 0.01^c^	3.58 ± 0.08^c^

Data are represented as Mean S.E.M (n = 4). Different superscript letters within rows in the same column indicate significant differences (p < 0.05) between different tested concentrations.

**Fig. 5:**
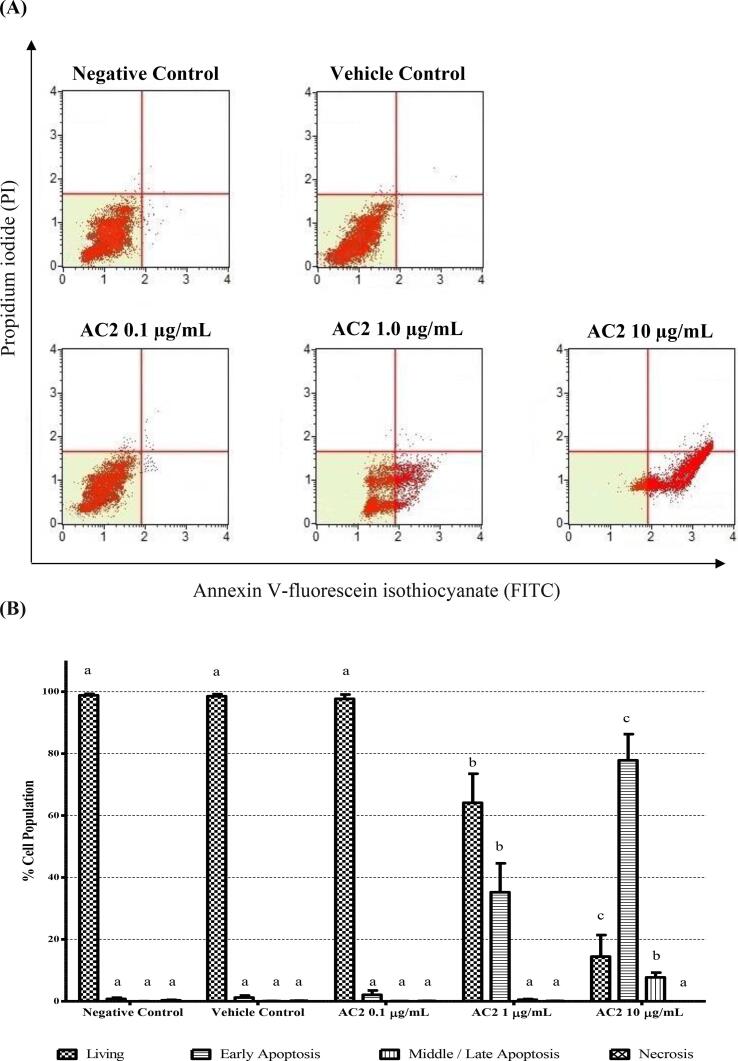
AC2 induced apoptosis in HUVECs at 24 h, as demonstrated by (A) quadrant graphs of cells treated with AC2 at various concentrations conducted by flow cytometry. (B) The cell apoptosis was also quantitatively analyzed (n = 3) and significant differences (p < 0.05) were indicated by different small letters between different experimental groups.

Following the inhibition of AC2 on HUVECs' proliferation, we investigated the possibility that AC2 induces programmed cell death using the ApopNexin^TM^ FITC Apoptosis Detection Kit. This kit contains Annexin V conjugated with FITC and propidium iodide (PI). In the negative control group, viable cells were non-fluorescent as their integrity was reported to be the majority population, as shown in Fig. 5A. Apoptotic cells, characterized by both early and late apoptotic cells, were observed in the lower-right and upper-right quadrants of the histograms, respectively. No significant comparison was observed between the vehicle control and 0.1 µg/mL AC2 with the untreated group, respectively (Fig. 5B).

The application of AC2 at a concentration of 1 µg/mL resulted in a notable decrease in the number of viable cells, with a reduction of 64.12 ± 9.36 %. Furthermore, the proportion of apoptotic cells increased to nearly 35 % of the total cell population. As the concentration of AC2 continued to increase, the population of HUVECs in early apoptosis grew at the expense of the viable population. Specifically, the early apoptosis count was reported to be 35 % at 1.0 µg/mL AC2 and increased to 2 times-fold at 10.0 µg/mL where a middle/late apoptosis population was observed. Notably, no necrotic cells were observed in any of the groups.

**Fig. 6:**
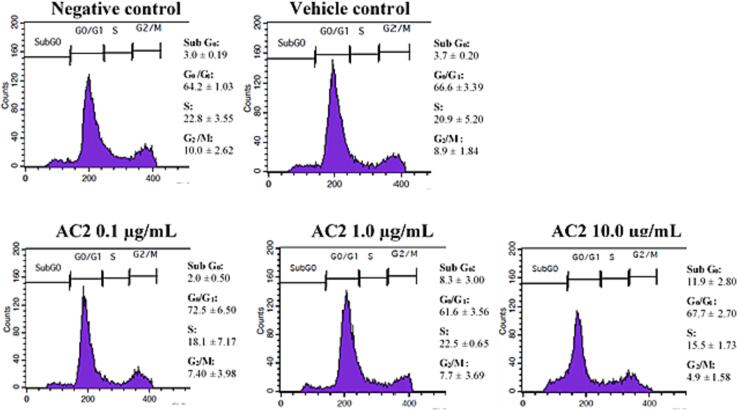
Cell cycle distribution on HUVECs treated with 0.1, 1.0 and 10 µg/mL AC2 for 24 h, respectively. The data is shown as % Mean ± SEM (n = 3).

In our subsequent evaluation of the impact of AC2 on HUVEC cell cycle distribution, we treated cells with AC2 and analyzed the results using a flow cytometric cell cycle assay. As presented in the representative flow cytometry histograms in Fig. 6, both the negative and vehicle controls exhibited a predominance of cells in the G_0_/G_1_ phase, with approximately 20.9–22.8 % of the cell population in this phase. The S phase occurred in about 8.9–10 % of the cells, followed by the G_2_/M phase in 3–10 % of the cells, and the G_0_ phase in 3–3.7 % of the cells.

Upon evaluation of the results, no significant differences in the G_0_/G_1_, S, and G_2_/M phases were observed between the treatment and negative control groups. However, we observed that AC2, at the highest concentration of 10 µg/mL, demonstrated a significantly higher sub G_0_ cell population compared to the negative control, relative to the G_0_/G_1_, S, and G_2_/M phase cell populations.

### 3.4 AC2 suppressed HUVECs migration, invasion, and differentiation

**Fig. 7:**
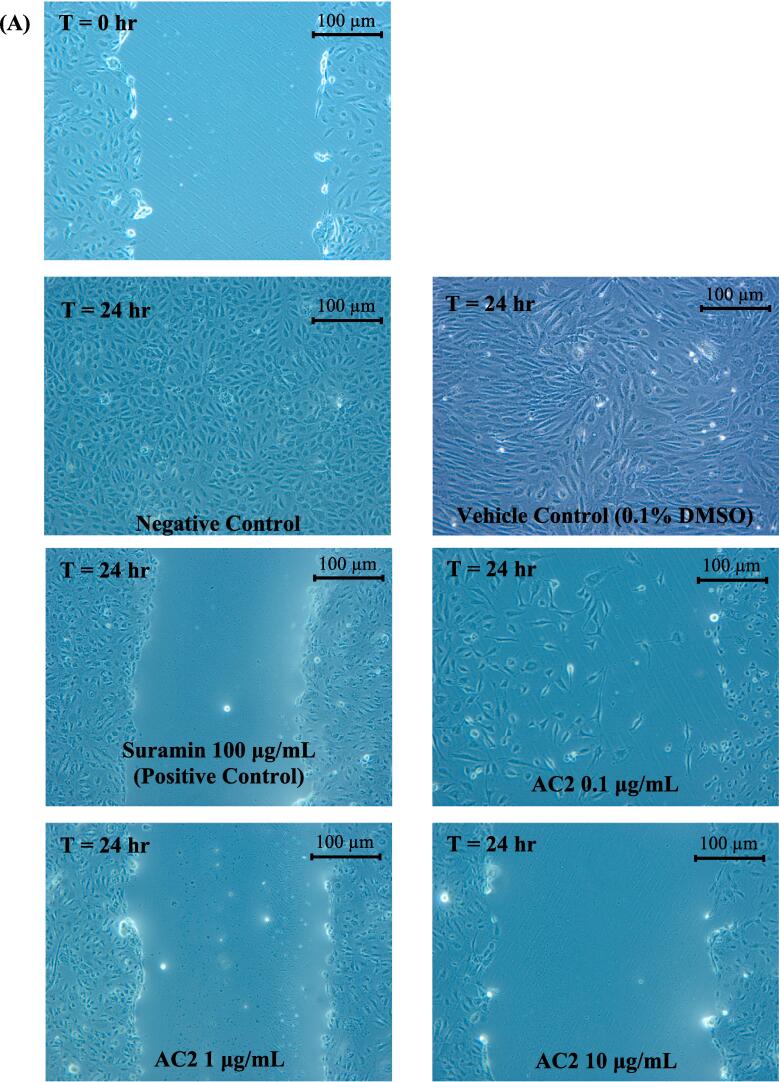

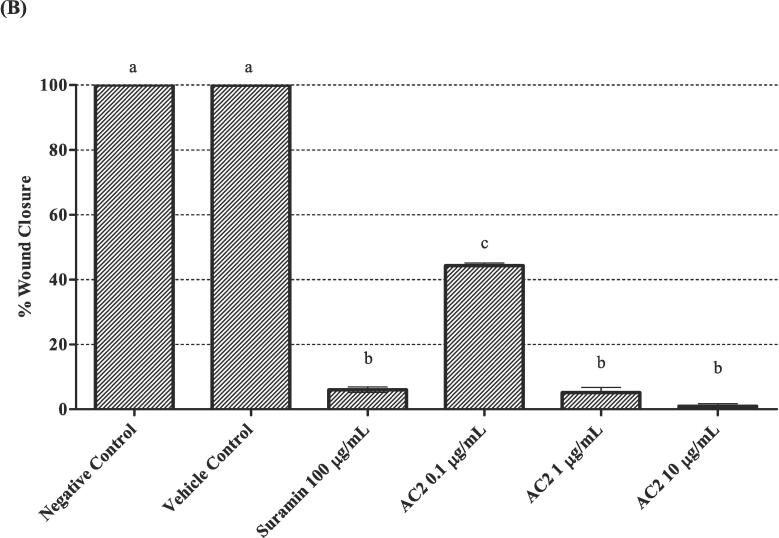
AC2 significantly impeded the migration of HUVECs after a 24-hour period at all concentrations tested. As demonstrated in the accompanying micrograph (A), taken at 0 and 24 h at 100 × magnification, the wounded area was observed to be significantly reduced in size. (B) Quantitative data (n = 3) supports this finding, with statistically significant differences observed between groups (p < 0.05, denoted by different small letters).

Endothelial cell migration to the perivascular area simulating the wound healing process *in vivo* is a key step in the tightly regulated angiogenesis process (Staton et al., 2009). Therefore, we assessed the motility of the endothelial cells treated with AC2 in the present study. The rapid migration of HUVECs from the edge of the scratches towards the wounded area was fully covered within 24 h in the negative and vehicle control groups (Fig. 7A). In the presence of AC2, HUVECs mobility was significantly decreased compared with that of the negative control, as shown in Fig. 7B. Motility retardation was significantly greater when AC2 concentration was increased to 1 μg/mL (*p* < 0.05). However, a further increase in AC2 concentration to 10 μg/mL did not significantly inhibit HUVEC migration. Interestingly, the inhibitory activity of AC2 at all tested concentrations was comparable to that of the positive control, suramin (*p* > 0.05).

Degradation of the basement membrane is stimulated by endothelial cells when activated by pro-angiogenic factors. An increasing gradient of chemoattractants promotes invasion and migration into the perivascular space (Adair & Montani, 2010). Chemotaxis is directional migration resulting from a gradient of stimuli, which differs from the migration of endothelial cells, which is solely due to random motility, known as chemokinesis.

**Fig. 8:**
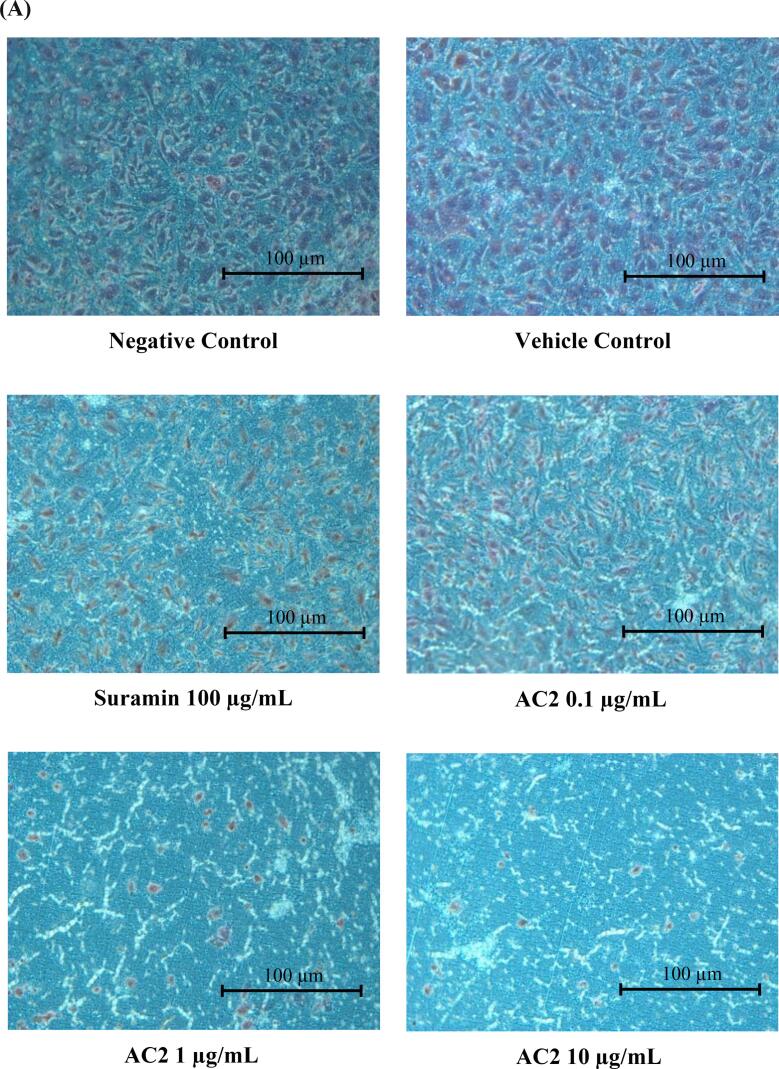

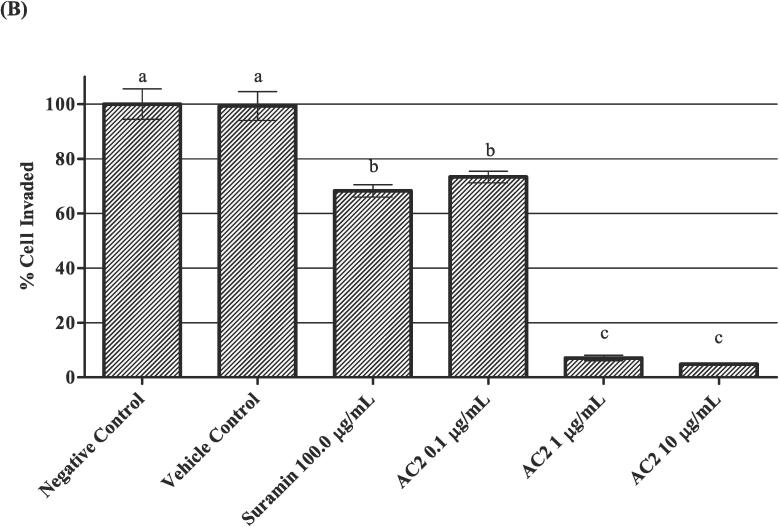
AC2 significantly decreased the invasion of HUVECs after 24 h, as shown by (A) micrographs with 200× magnification and purple-stained nuclei (B) Quantitative data (mean ± SEM, n = 3) supports this finding, with significant differences between experimental groups indicated by (p < 0.05, different small letters).

No significant difference was observed between the negative control and vehicle control groups, which justified the lack of influence of the vehicle (0.1 % DMSO) on promoting any effects in the current experiment (Fig. 8A). Generally, AC2 at 0.1, 1.0, and 10 µg/mL significantly attenuated the number of invading cells compared to that in the negative control group (*p* < 0.05) (Fig. 8B). In fact, AC2 at 1 and 10 µg/mL respectively altered the cell invasion to 7.09 ± 1.03 % and 4.80 ± 0.34 %, respectively. At the lowest concentration, AC2 was still able to suppress cell invasion, which was comparable to that of the positive control group.

Upon stimulation by angiogenic inducers, endothelial cells proliferate and move towards the proangiogenic chemoattractant by invading the perivascular space. The differentiation of HUVECs from tube-like structures consists of a lumen surrounded by endothelial cells linked through junctional complexes. (Goodwin, 2007, Adair and Montani, 2010). Similarly, HUVECs are induced to differentiate and form tubules from its characteristic ‘cobblestone’ morphology when cultured on a matrix of basement extract (BME) such as Matrigel. Therefore, we assessed the ability of AC2 to inhibit HUVEC differentiation using a tube-formation assay in the current study.

**Fig. 9:**
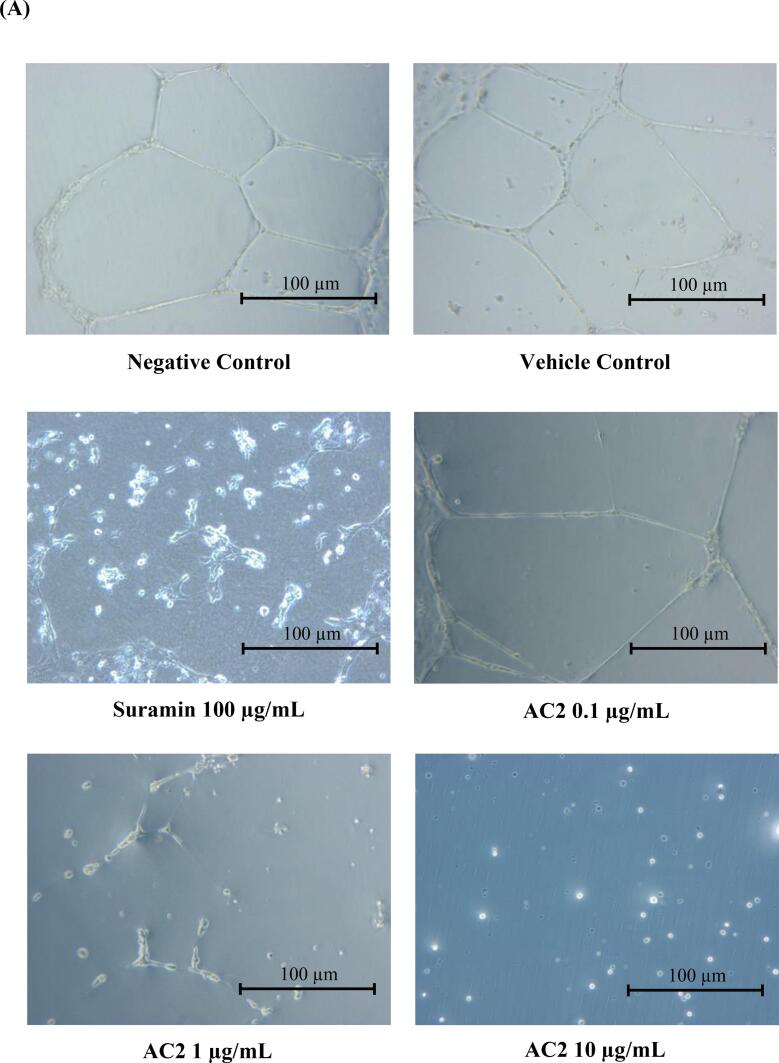

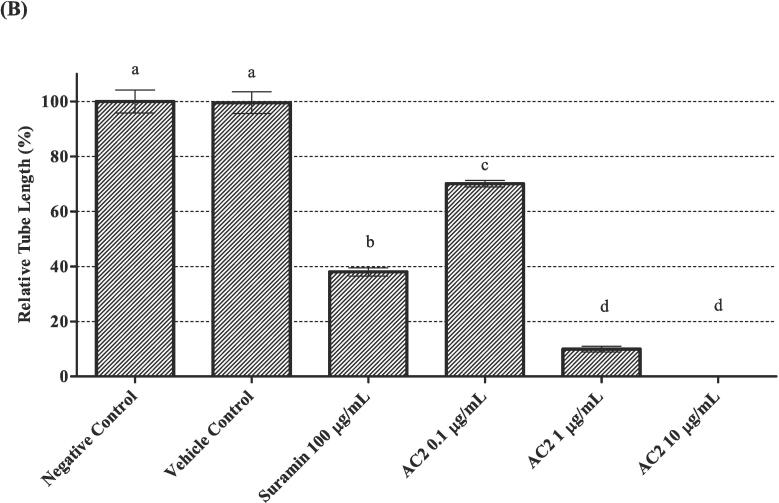
AC2 significantly disrupted HUVEC tubular network formation (A) Illustrated micrographs showed HUVECs tubule formation was inhibited by AC2 after 16 h of treatment (200× magnification). (B) Quantitative data was measured, and the negative control was normalized to 100 %. Significant differences (p < 0.05) between experimental groups are indicated by different small letters.

The negative and vehicle control groups exhibited excellent capillary tubular networks. Conversely, after 16 h of treatment, AC2 showed an impressive disruption of tubular network formation at all concentrations (Fig. 9A). Approximately one-third of HUVEC differentiation was significantly inhibited, even at the lowest concentration of AC2. Similarly, tubule formation was also inhibited at higher concentrations (Fig. 9B).

### 3.5 AC2 significantly altered multiple proangiogenic protein expressions, in vitro

During the initial stage of angiogenesis, activated endothelial cells release various proteases such as MMPs to degrade and remodel the extracellular matrix (ECM) and basement membrane. In the later stages of angiogenesis, it can ease the proliferation and migration of endothelial cells (Toth et al., 2012). MMP-2 and MMP-9 can degrade type IV collagen, gelatin, laminin, and elastin, which are the major components of the basement membrane (Frankowski et al., 2012). AC2 has previously shown significant reduction of HUVEC invasion and tubule formation, thus the possible association of MMPs in the antiangiogenic effect of AC2 was further investigated.

**Fig. 10:**
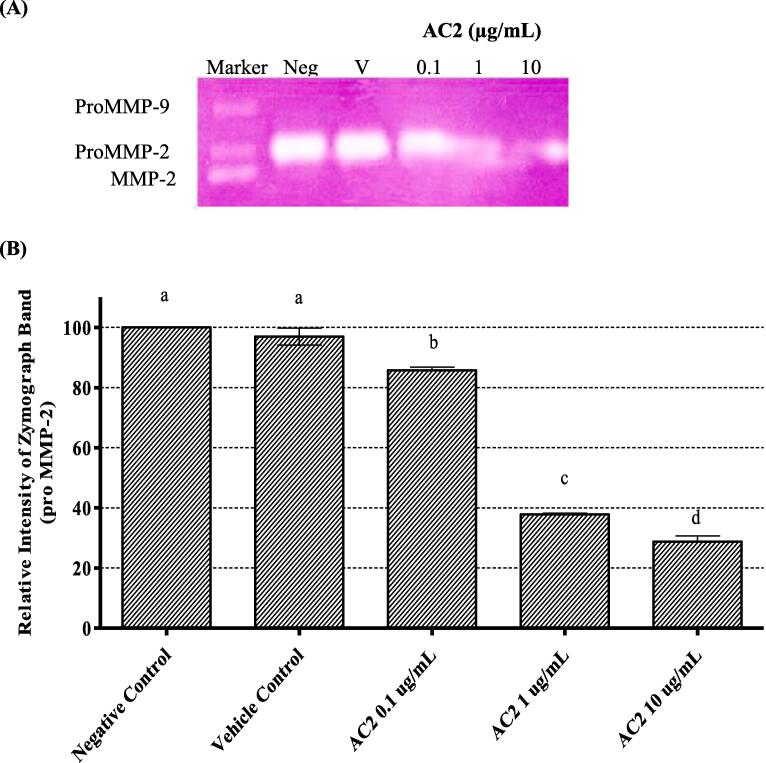
AC2 significantly decreased MMP activity, as demonstrated by (A) a gelatin zymography gel displaying a gradient of proMMP2 band density with increasing concentrations of AC2. (B) Quantitative data are presented, with the negative control normalized to 100 %; the mean ± SEM (n = 3) is shown. Significant differences (p < 0.05) between experimental groups are indicated by different small letters.

In this study, gelatinase activity was determined by examining the conditioned culture media of HUVECs treated with various concentrations of AC2 for 24 h. As depicted in Fig. 10A, only proMMP-2 was observed in the gel. However, MMP-2 and proMMP9 expressions were absent in the culture medium. Based on these results, AC2 (1 and 10 µg/mL) significantly reduced proMMP-2 secretion in a concentration-dependent manner (*p* < 0.05) (Fig. 10B). Thus, it is suggested that the anti-angiogenic activity of AC2 is partly due to the inhibition of proMMP-2 secretion.

Possible angiogenesis protein markers involved in the anti-angiogenic effects of AC2 were evaluated via a multiplex assay, which only produced 10 relevant results out of 17 analytes from the panel (EMD Millipore, USA).

**Fig. 11:**
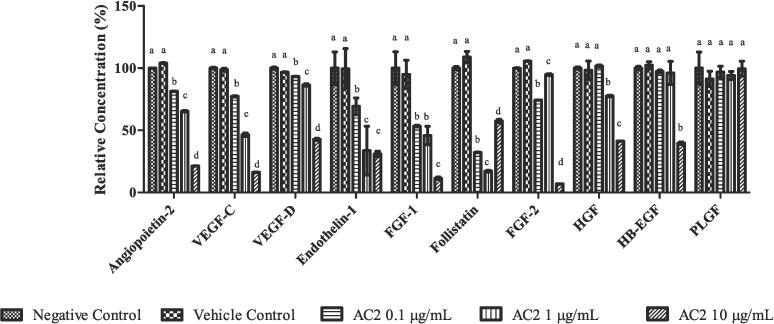
AC2 caused a notable decrease in the levels of multiple proteins, as determined by the mean ± SEM (n = 3), with normalization of the negative control to 100 %. Different small letters indicate significant differences (*p* < 0.05) between different treatment groups in the same protein expression.

The treatment of HUVEC lysates with AC2 at all concentrations significantly and concentration-dependently reduced (*p* < 0.05) the expression of Angiopoietin-2, VEGF-C and VEGF-D (Fig. 11). The expression of ENT-1 (Endothelin-1) showed significant reduction (*p* > 0.05) at all concentrations, with the 1 and 10 µg/mL treatment groups exhibiting an equal reduction. Similarly, AC2 at 0.1 and 1 µg/mL significantly reduced the expression of FGF-1, with the highest reduction observed at its highest concentration (10 µg/mL).

Both Follistatin and FGF-2 were significantly and concentration-independently reduced (*p* < 0.05) in AC2 at all concentrations. The reduction of follistatin expression was concentration dependent from 0.1 µg/mL (32.231 ± 0.47 %) to 1 µg/mL (17.166 ± 1.016 %), when it was significantly increased (54.46 ± 2.293 %) when the concentration of AC2 increased to 10 µg/mL. Conversely, the expression of FGF-2 was significantly increased from 74.319 ± 0.249 % (0.1 µg/mL) to 84.832 ± 1.158 % (1 µg/mL) then drastically reduced to 6.922 ± 0.329 % when AC2 was increased to 10 µg/mL.

A significant reduction in HGF expression was observed at 1 and 10 µg/mL AC2, in a concentration-dependent manner. Moreover, AC2 at the highest concentration (10 µg/mL) significantly attenuated (*p* < 0.05) the expression of HB-EGF in the HUVEC lysates. PLGF expression was not significantly different between the groups.

### 3.6 AC2 inhibited *in vivo* angiogenesis on zebrafish embryo intersegmental vessels (ISV)

**Fig. 12:**
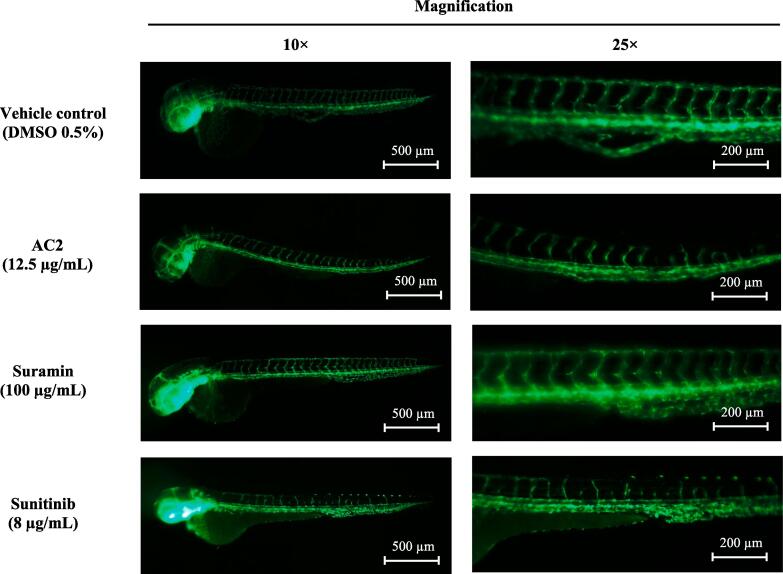
AC2 displayed partial inhibition of neovascularization in a zebrafish embryo model. The micrographs depict transgenic *Tg(fli1:EGFP)^y1^* zebrafish embryos incubated for 24 h with various treatments at 18 h post-fertilization (hpf), and stained with an EGFP tag.

As most in vitro angiogenesis assays were successfully inhibited by AC2, AC2 was further assayed in zebrafish embryos *in vivo* to determine its antiangiogenic effect. At 24 h post-treatment, all embryos were nonviable at 25 µg/mL AC2. At 12.5 µg/mL, AC2 significantly inhibited ISV in embryos (Fig. 12, Table 4). Moreover, AC2 partially inhibited dorsal longitudinal anastomotic vessel (DLAV) formation in embryos. Nonetheless, at higher concentrations, AC2 did not show any inhibitory effect on ISV sprouting. On the other hand, the positive control, an established antiangiogenic compound, sunitinib (8 µg/mL) totally inhibited ISV sprouting of the embryos and none can be seen on suramin (Table 4).

**Table 4:** Antiangiogenic effect of AC2 in zebrafish.

Treatment	Number of embryos with inhibited ISV sprouting*			Embryos with inhibited ISV sprouting (%)
	^#^Complete	^##^Partial	^###^Minor	
Vehicle control	0	0	0	0
AC2 12.5 µg/mL	0	2	3	25
Suramin 100 µg/mL	0	0	0	0
Sunitinib 8 µg/mL	20	0	0	100

*Total sample = 20; ^#^Complete inhibition of ISV sprout; ^##^Inhibition of ≥ 4 ISV sprout; ^###^.

## 4 Discussion

The importance of antiangiogenic therapy in treating angiogenesis-associated diseases, including cancer and Alzheimer’s disease, has been well publicized. Natural compounds serve as potential angiogenesis modulators that can minimize drugs and their side effects, thus providing a wide range of potential research, which may lead to novel therapies (Ribeiro et al., 2018). Although several antiangiogenic drugs, such as sorafenib, vandetinib, and suntinib, have been clinically used in the treatment of various cancers, a major problem may occur later due to drug resistance. In addition, similar to other chemotherapeutic drugs, the toxicity of these antiangiogenic drugs contributes to side effects, leading to serious injuries (Gotink and Verheul, 2010). Therefore, researchers should develop new antiangiogenic agents with less toxicity and the ability to treat and prevent pathological angiogenesis (Lewandowska et al., 2014). Natural antiangiogenic molecules have been in the limelight for decades because of their accessibility, low toxicity, and, most importantly, their traditional uses in the treatment of multiple diseases (Wang et al., 2015). In this case, curcumin and Epigallocatechin-3-gallate (EGCG) are 2 natural compounds cum nutraceuticals been reported for their antiangiogenic activities (Shakeri et al., 2019, Domingo et al., 2010).

Natural compounds with anti-angiogenic properties are mostly composed of phenolic compounds because of their high affinity to diverse molecular targets (Pascual-Teresa et al., 2004). Quinones, a class of phenolic compounds, have been reported to possess various pharmacological properties and have many applications in the fields of pharmacy and medicine. Quinonoid compounds are derived from the skeleton of 1,4-benzoquinone, also known as *p*-benzoquinone. In fact, many chemical derivatives with a basic structure consisting of 1,4-benzoquinone have been reported to exhibit various pharmacological effects, such as antibiotic (Koyama, 2006) and antitumor effects (Silva et al., 2009).

In this study, we report the anti-angiogenic activities of a benzoquinonoid compound, AC2, in human umbilical vein endothelial cells (HUVECs). 2-methoxy-6-tridecyl-1,4-benzoquinone, a compound with a chemical structure similar to that of AC2, has been shown to hinder tumor growth in animal models and prevent the metastasis of cancer cells to the lungs (Kang et al., 2001). Given the close relationship between angiogenesis and chronic inflammation, it is important to explore AC2′s antiangiogenic effects. Angiogenesis plays a critical role in metastasis.

AC2 has been demonstrated to exhibit a stronger antiproliferative effect against HUVECs after incubation for 24, 48, and 72 h, although this effect was not time dependent. The IC_50_ values were determined to be 1.35 ± 0.046, 1.15 ± 0.017, and 1.00 ± 0.012 µg/mL, respectively. At concentration of 2.5 µg/mL and above, AC2 was found to be cytostatic, with a cell viability of approximately 6 %. However, the antiproliferative effect of AC2 was not due to cytotoxicity, as even at concentrations of 0.1 and 1 µg/mL, more than 50 % of the cells remained viable (Table 3).

Several studies have shown that endothelial cell apoptosis potentially limits angiogenesis, which, in turn, leads to blood vessel regression in adult neovascularization ((Dimmeler and Zeiher, 2000). During apoptosis, endothelial cells tend to prevent nutrient supply by hindering new blood vessel formation in tumor tissues, emphasizing the importance of apoptotic mechanisms in prevention and tumor therapies (Park et al., 2007). Interestingly, AC2 significantly inhibited HUVECs migration, invasion, and differentiation at all concentrations tested (Fig. 8B–Fig. 10B), which clarified its antiangiogenic effects, as the aforementioned assays are important determinants of anti-angiogenic effects. Even at lower concentrations, AC2 was still able to significantly suppress the angiogenic effects, which confirmed the antiangiogenic potential of the compound.

The anti-invasive activity of AC2 was confirmed by the suppression of HUVECs invasion by AC2 at all concentrations, even at noncytotoxic concentrations (Fig. 8B). These results indicate the ability of AC2 to inhibit endothelial cell invasion and raise the possibility that AC2 may block endothelial invasion, presumably by inhibiting of matrix metalloproteinases (MMPs) activity.

MMPs play a crucial role in ECM degradation and are involved in promoting endothelial and tumor invasion (Stetler-Stevenson, 1999). Endothelial cells produce several MMPs that regulate angiogenesis, including MMP-2 and MMP-9. MMP-2 is secreted and activated by invasive endothelial cells. In this study, we examined the correlation between the anti-invasiveness of AC2 and inhibition of gelatinolytic MMPs activity in HUVECs using a zymogram assay. We found that the pro-MMP2 band was prominent in the gelatinolytic bands of both vehicle and untreated cells, while the pro-MMP9 band was barely detectable. The MMP2 band was not detectable in HUVECs that were not induced by any growth factors, because pro-MMP2 could not be activated by MMP2 (Kim et al., 2000, Chun et al., 2004). AC2 inhibits the proteolytic activation of proMMP2 in a concentration-dependent manner, possibly by directly activating pro-MMP2, which blocks the invasiveness of endothelial cells.

Several proangiogenic factors are inhibited by AC2 to varying degrees. AC2 significantly reduced the expression levels of Angiopoeitin-2, VEGF-C and VEGF-D in a concentration-dependent manner (Fig. 12). Although Ang-2 has been reported to act as an angiogenesis inhibitor, it has a dual function under certain conditions. Overexpression of Ang-2 induces endothelial cell migration and tube formation in vitro (Felcht et al., 2012), thus clarifying the role of this growth factor in the effect of AC2 in inhibiting HUVECs migration and differentiation. VEGF-C and VEGF-D are lymphangiogenic growth factors because of their ability to induce tumor lymphangiogenesis, which directly metastasizes to lymphatic vessels and lymph nodes by binding to the vascular endothelial growth factor receptor 3 (VEGFR-3), thus promoting lymphangiogenesis. VEGFR-3 is necessary for the development of lymphatic vessels by promoting proliferation, survival, and migration of lymphatic endothelial cells (LECs) (Stacker et al., 2002). Interestingly, our current findings demonstrated the suppression of both VEGF-C and VEGF-D by AC2, which might also correlate with our previous findings on ACRH and QRF, containing AC2 which demonstrated antitumor promotion in chemically induced skin tumorigenesis (Yeong et al., 2013, Yeong et al., 2015). This is in accordance with the findings of Alitalo et al. (2013), who reported that blockade of VEGF-C and VEGF-D also inhibited skin inflammation by targeting early-stage tumor growth. In addition, the anti-invasive activity of AC2 observed in the cell invasion assay may be attributed to the suppression of VEGF-C and VEGF-D by the compound. Although VEGF-A could not be measured in the current study, our group demonstrated the suppression of VEGF-A in *an in vivo* arthritic animal study (Blin et al., 2021a) treated with QRF, which might occur in the effect of AC2 as well.

Generally, FGFs are required as the modulator in the endothelial cells’ proliferation and migration, and also in producing proteases, and promoting expression of integrin and cadherin receptor (Javerzat et al., 2002). In tumor angiogenesis, FGF-1 and FGF-2 promote endothelial cell proliferation. They are also important for the growth of new blood vessels at the excision wound site and can stimulate angiogenesis in various assays (Yang et al., 2015). Our current findings demonstrated that both FGF-1/2 were significantly inhibited by AC2, which may be responsible for the inhibition of HUVECs proliferation, migration, and tubule formation by AC2 in these assays.

Endothelin-1(ENT-1) is a potent protease (Salani et al., 2000). Therefore, suppression of endothelin-1 by AC2 may have contributed to the suppression of HUVECs proliferation, migration, invasion, and pro-MMP2 production observed in the current study. Moreover, endothelin-1 may also play important role in our previous findings, in which ACRH and QRF (consists of AC2) suppressed the vascularization in *in vivo* Miles vascular permeability and murine air-pouch granuloma assays (Hamsin et al., 2013, Nidavani et al., 2014).

*In vitro*, follistatin (FST) was also been reported to play an essential role in endothelial cell migration, tubule formation, and sprouting in expanding the vasculature (Fahmy-Garcia et al., 2019). Hence, this is in line with the suppression of tube formation by AC2, which was mediated by the suppression of the FST in our findings.

HGF is required for the induction of endothelial cell degradation of the ECM, migration, and proliferation (Bussolino et al., 1992, Ma et al., 2002). In addition, HGF has been reported to be antiapoptotic in HUVECs (Ma et al., 2002). Therefore, HGF suppression may have contributed to the suppression of pro-MMP2 in the zymogram assay and the apoptotic effect of AC2 in the current study as well as its migratory inhibition and antiproliferative effects on HUVECs. Because HGF is overexpressed in human atherosclerotic plaques (Ma et al., 2002), it is necessary to conduct future study on AC2′s potential in atherosclerosis.

However, HB-EGF was significantly inhibited at higher concentrations of AC2 (10 µg/mL). Mehta and Besner (2007) reported the role of HB-EGF and EGF in stimulating and promoting migration and vascular tube formation in HUVECs, respectively, without inducing cell proliferation. Moreover, the inhibition of the p38 MAPK pathway enhances the migration and angiogenesis of endothelial cells induced by HB-EGF (Mehta et al., 2008). Therefore, we postulated that at higher concentrations, suppression of HB-EGF may also contribute to the anti-migratory and tube formation inhibitory effects of AC2 in the current study, and this may be mediated via the PI3 kinase and MAPK pathways (Mehta and Besner, 2007).

Currently, the use of zebrafish as a valuable model organism to substitute traditional models for assessing potential antiangiogenic agents is well established. Angiogenesis simulation in cancer can mimic the formation of blood vessels in zebrafish embryos. Antiangiogenic indicators can be evaluated by observing intersegmental vessels (ISVs) and subintestinal veins (SIVs) in zebrafish embryos (Zhang et al., 2018). In this study, we investigated the effects of AC2 using a zebrafish model. Our current findings showed the anti-angiogenic effect of AC2 by partially inhibiting ISV sprouting in zebrafish embryos (25 %) at a slightly higher concentration (12.5 ug/mL) compared to in vitro assays. However, an increased concentration of AC2 was toxic to zebrafish embryos. Further studies on zebrafish embryos are suggested by conducting TUNEL staining on fli1:EGFP background embryos to determine if the anti-angiogenic effects are due, at least in part, to endothelial-specific apoptosis (as observed in HUVECs).

## 5 Conclusion

2-methoxy-6-undecyl-1,4-benzoquinone (AC2), isolated from the root of *Ardisia crispa*, has potential as an anti-angiogenic agent, as demonstrated by its inhibitory effect on HUVECs in various angiogenesis assays. Moreover, high concentrations of AC2 induce apoptosis. In vivo studies in zebrafish have also revealed a promising inhibition of angiogenesis. AC2 significantly suppressed the expression of essential angiogenesis and protein markers of growth factors including proMMP2, VEGF-C, VEGF-D, Angiopoietin-2, FGF-1, FGF-2, Endothelin-1, Follistatin, HGF, and HB-EGF. Further investigation is required to elucidate its mechanism(s) and effect on other excessive angiogenesis diseases, such as various types of cancer and atherosclerosis.

## Funding

This work was funded by the 10.13039/501100003093Ministry of Higher Education, Malaysia, under the Explorative Research Grant Scheme (ERGS) with Project no: ERGS/1-2013/5527165.

## Declaration of generative AI and AI-assisted technologies in the writing process

During the preparation of this study, the author(s) used PAPERPAL to improve language and readability with caution. After using this tool/service, the author(s) reviewed and edited the content as needed and took (s) full responsibility for the content of the publication.

## CRediT authorship contribution statement

**Wen Jun Lim:** Investigation, Visualization, Data curation, Writing – original draft. **Pit Foong Chan:** Visualization, Investigation. **Roslida Abd Hamid:** Conceptualization, Methodology, Supervision, Validation, Writing – review & editing.

## Declaration of competing interest

The authors declare that they have no known competing financial interests or personal relationships that could have appeared to influence the work reported in this paper.
